# Effectiveness of ALK inhibitors in treatment of CNS metastases in NSCLC patients

**DOI:** 10.1080/07853890.2023.2187077

**Published:** 2023-03-10

**Authors:** Michał Gil, Magdalena Knetki-Wróblewska, Przemysław Niziński, Maciej Strzemski, Paweł Krawczyk

**Affiliations:** a Department of Pneumonology, Oncology and Allergology, Medical University of Lublin, Lublin, Poland; b Department of Lung Cancer and Chest Tumors, Maria Sklodowska-Curie National Research Institute of Oncology, Warsaw, Poland; c Department of Pharmacology, Medical University of Lublin, Lublin, Poland; d Department of Analytical Chemistry, Medical University of Lublin, Lublin, Poland

**Keywords:** Non-small cell lung cancer, anaplastic lymphoma kinase, blood-brain barrier, ALK inhibitors

## Abstract

Metastases to the central nervous system (CNS) in patients with non-small cell lung cancer constitute an extremely difficult clinical problem, and their occurrence is associated with a poor prognosis. Due to the existence of the blood-brain barrier (BBB) and the action of proteins responsible for the transport of drugs, e.g. P-glycoprotein (P-gp), the penetration of drugs into the CNS is insufficient. Until recently, the only method of CNS metastases treatment was radiotherapy and neurosurgery. The advancement of molecular biology allowed discover targets for molecularly targeted therapies. One of targets is abnormal anaplastic lymphoma kinase, which results from the rearrangement of the *ALK* gene in patients with non-small cell lung cancer (NSCLC). *ALK* rearrangement occurs in only about 4.5% of NSCLC patients, but its presence favors brain metastases. The ALK inhibitors (ALKi) were modified to obtain molecules with high ability to penetrate into the CNS. This was achieved by modifying the structure of individual molecules, which became, inter alia, less substrates for P-gp. These modifications caused that less than 10% of patients experience progression in CNS during new ALK inhibitors treatment. This review summarizes the knowledge about the action of BBB, the pharmacodynamics and pharmacokinetics of ALKi, with particular emphasis on their ability to penetrate the CNS and the intracranial activity of individual drugs from different generations of ALK inhibitors.

## KEY MESSAGES

*ALK* gene rearrangement favors the development of central nervous system (CNS) metastases in patients with NSCLC. New generations of ALK inhibitors penetrate the CNS and induce an intracranial response and protect against new brain lesions.

## 1. Introduction

*ALK* (anaplastic lymphoma kinase) gene rearrangement occurs in approximately 4.5% of non-small cell lung cancer (NSCLC) patients. It is observed almost exclusively in patients with adenocarcinoma. It occurs more often in young, non-smoking patients and in women. The most common fusion partner for the *ALK* gene is the *EML4* gene (echinoderm microtubule-associated protein-like 4). Fusions of exon 20 of the *ALK* gene with exons 13 (Variant 1), 20 (Variant 2) or 6 (Variant 3) of the *EML4* gene are the most frequently observed. However, other fusion variants and non-EML4 fusion partners exist. Understanding the molecular backgrounds of *ALK* gene rearrangement has been made possible thanks to the wider use of the next-generation sequencing (NGS) technique, which is slowly replacing monogeneous techniques such as fluorescent *in situ* hybridization (FISH) and immunohistochemistry (IHC) techniques in routine genetic diagnosis of NSCLC patients [1].

Rearrangement of the *ALK* gene leads to the formation of a protein with the activity of tyrosine kinase located entirely inside the tumor cell (no transmembrane and extramembrane parts of the ALK receptor). This activates the kinase in a ligand-independent manner and results in the proliferation and inhibition of apoptosis of tumor cells [2]. The abnormal ALK protein can be effectively blocked by ALK inhibitors of which three generations have been developed: first generation (crizotinib), second generation (ceritinib, alectinib, brigatinib, ensartinib) and third generation (lorlatinib). Although crizotinib is still registered for the first-line treatment of patients with locally advanced or advanced NSCLC, it is highly preferable to start therapy with second- or even third-generation drugs [3].

In embryogenesis, the normal ALK protein is involved in the development of the central nervous system (CNS). Therefore, the occurrence of *ALK* gene rearrangement in NSCLC patients promotes the metastasis of lung cancer to the CNS. It is estimated that at the time of NSCLC diagnosis, 20% of patients with *ALK* gene rearrangement have brain metastases. Further, 40–50% of such patients will develop CNS metastases during the disease course. Anti-cancer therapies are rarely effective in CNS metastates tretament due to the existence of the blood-brain barrier (BBB). Therefore, it has become extremely important to develop new drugs that penetrate the CNS. These molecules should have the ability to treat existing metastases and to prevent new metastases formation [4]. The next generation of ALK inhibitors show such properties.

The assessment of intracranial activity of ALK inhibitors is of key importance when the value of the therapy is analysed. Considering that many patients are relatively young and active, it is important to achieve optimal disease control in this area, to defer brain irradiation along with the potential side effects of this treatment. Due to cognitive impairment and functional deterioration after the treatment, SBRT (stereotactic body radiation therapy) rather than WBRT (whole brain radiation therapy) is recommended. In selected clinical situations – in case of the presence of a lesion threatening to wedge – neurosurgery may be a treatment of choice. However, multiple secondary lesions are usually present, often without significant clinical signs. In such cases, the effective systemic treatment appears to be an optimal choice [5]. Drugs differ in their ability to penetrate the blood-brain barrier and thus have different antitumour potential in this area.

## 2. Blood-brain barrier

The blood-brain barrier consists of 3 components: endothelial cells of capillaries, astrocytes and pericytes. Endothelial cells are specialized epithelium lining the lumen of a capillary vessel in the brain [6]. This epithelium has unique features, i.e. no fenestration, a large number of mitochondria due to high cellular metabolism, low pinocytic activity, the presence of tight junctions and selective permeability to molecules [6,7]. Astrocytes mediate between capillaries and neurons in the brain. Their parts additionally protect the brain against substances that have passed through endothelial cells. The final component are pericytes, small cells that surround the endothelial cells of the epithelium. They control immunological processes, have the ability to phagocytose and control the diameter of capillaries [6,8].

The integrity of the BBB corresponds to 2 types of cells connections. Tight-type junctions are composed of occludins, claudins, and cell adhesion proteins as well as proteins associated with the actin cytoskeleton. Occludins are a 60 kDa phosphoprotein that regulates transmembrane transport by maintaining an appropriate electrical resistance. Claudins are a family of 24 proteins that build tight junctions [6,7,9]. Cell adhesion proteins are immunoglobulins. The cytoplasmic proteins zonula occludens are the most important proteins in this type of junction, they bind occludins, claudins and adhesion proteins to the actin cytoskeleton. The second type of connections are adherence bonds in which the main components are catherins [6–8,10]. The blood-brain barrier has many functions. It allows to maintain the appropriate concentration of ions for the proper functioning of neurons and protects the brain from the excessive activity of neurotransmitters. The BBB performs nutritional functions through numerous transport channels and protects the brain from the action of exo- and endogenous toxins, such as harmful metabolites, drugs or xenobiotics. It also stops most of the proteins from reaching the brain [6,7,10].

The transport of substances by the BBB is mainly based on the principle of active transport. Transporters such as GLUT-1 (glucose transporter 1), Na/K pump and AQP1 (aquaporin-1) water transporter are located in the membrane. Gases and fat-soluble substances pass through the BBB by simple diffusion. These mechanisms make it possible to control substances reaching the brain and maintain homeostasis [9].

## 3. Mechanisms of drugs elimination from CNS

ABC transporters (ATP-binding cassette) occur on the luminal side of the endothelial cells of the brain’s capillaries. It is a family of transporters of about 48 transport proteins divided by the Human Genome Organization (HUGO) into 7 classes ABC-A to ABC-G. Among them, we can distinguish importers and exporters [11–13]. The most important function of these transporters is the efflux of the molecules from the cells. For this process, they use energy from ATP. The most important of ABC transporters are the ABCB1 (ATP-binding cassette subfamily B member (1) otherwise known as P-glycoproteins (P-gp) and ABCG2 (ATP-binding cassette subfamily G member (2) also known as breast cancer resistance proteins (BCRP) [14,15]. They are responsible for the development of multi-drug resistance by efflux chemotherapeutic and molecularly targeted agents outside the cells and by inhibiting their delivery to the proper organ [14,16–20].

P-glycoprotein also called multi-drug resistance 1 (MDR1) is encoded by the *MDR* gene located on the long arm of chromosome 7. P-gp is the most abundant transport protein in human cells, including the cells in the blood-brain barrier. It is a 170 kDa protein, consisting of 2 homologous halves with 6 hydrophobic domains and an ATP binding site. It has a cylindrical shape and a channel through which it transports hydrophobic, inert substances and cations. It has a four-domain structure, with two nucleotide-binding domains (NBDs) each preceded by a polytopic membrane-spanning domain (MSD) composed of six transmembrane (TM) helices (MSD-NBD MSD-NBD). P-gp has a wide range of substrates [14,15,19].

The BCRP/ABCG2 protein is encoded by the *BCRP* gene located on chromosome 4. BCRP/ABCG2 is an atypical, so-called half-transporter, consisting of a single hydrophobic MSD which contain 6 TM helices preceded by a single NBD (NBD MSD). This protein is present on many cells, including the endothelial cells of the brain’s capillaries [14,19].

Both proteins are responsible for the phenomenon of multi-drug resistance. Physiologically, they protect the brain from harmful substances, xenobiotics and toxins. However, in the case of pharmacotherapy, they reduce the penetration of the drug into the organ. This makes it necessary to administer a higher dose to increase the concentration of the drug in the brain, which causes more side effects. The use of molecules that are not substrates for P-gp and BCRP is also a way to increase the concentration of drugs in cancer cells and penetrate them through the BBB [14,17].

## 4. ALK inhibitors – structure, physicochemical and pharmacological properties

In the treatment of advanced or locally advanced NSCLC patients with ALK rearrangement, the first approved targeted small-molecule TKI was crizotinib. Due to the observed resistance for its anti-neoplastic activity, and on the other hand, poor brain-blood barrier penetration it was necessary to develop new TKIs. Second-generation ALK inhibitors such as ceritinib, alectinib and brigatinib were proposed as more efficient in controlling brain metastases. Despite the achievement of better therapeutic results, the problem of ALK-resistance still remains [21]. Third-generation ALK inhibitors including lorlatinib seem to be effective even after the failure of the usage of other ALK inhibitors [22,23] (Table 1).

**Table 1.** Selected physicochemical properties of ALK inhibitors and compliance with the rules of Lipiński and Veber [28,29].

No.	ALK inhibitor	Molecular weght	LogP*	HBDC	HBAC	RBC	tPSA [Å^2^]	Fulfilling Lipinski’s rule	Fulfilling Veber’s rule
1	Alectinib	482.6	5.2	1	5	3	72.4	−	+
2	Brigatinib	584.1	4.6	2	9	8	85.9	−	+
3	Ceritinib	558.1	6.4	3	8	9	114	−	+
4	Crizotinib	450.3	3.7	2	6	5	78	+	+
5	Ensartinib	561.4	3.6	3	8	6	123	−	+
6	Lorlatinib	406.4	1.5	1	7	0	110	+	+

LogP: octanol-water partition coefficients; HBDC: Hydrogen Bond Donor Count; HBAC: Hydrogen Bond Acceptor Count; RBC: Rotatable Bond Count; tPSA: topological Polar Surface Area.

* Computed by XLogP3 3.0 (PubChem release 2021.05.07). The red color indicates values that do not satisfy the Lipinski rule.

**Figure 1.**
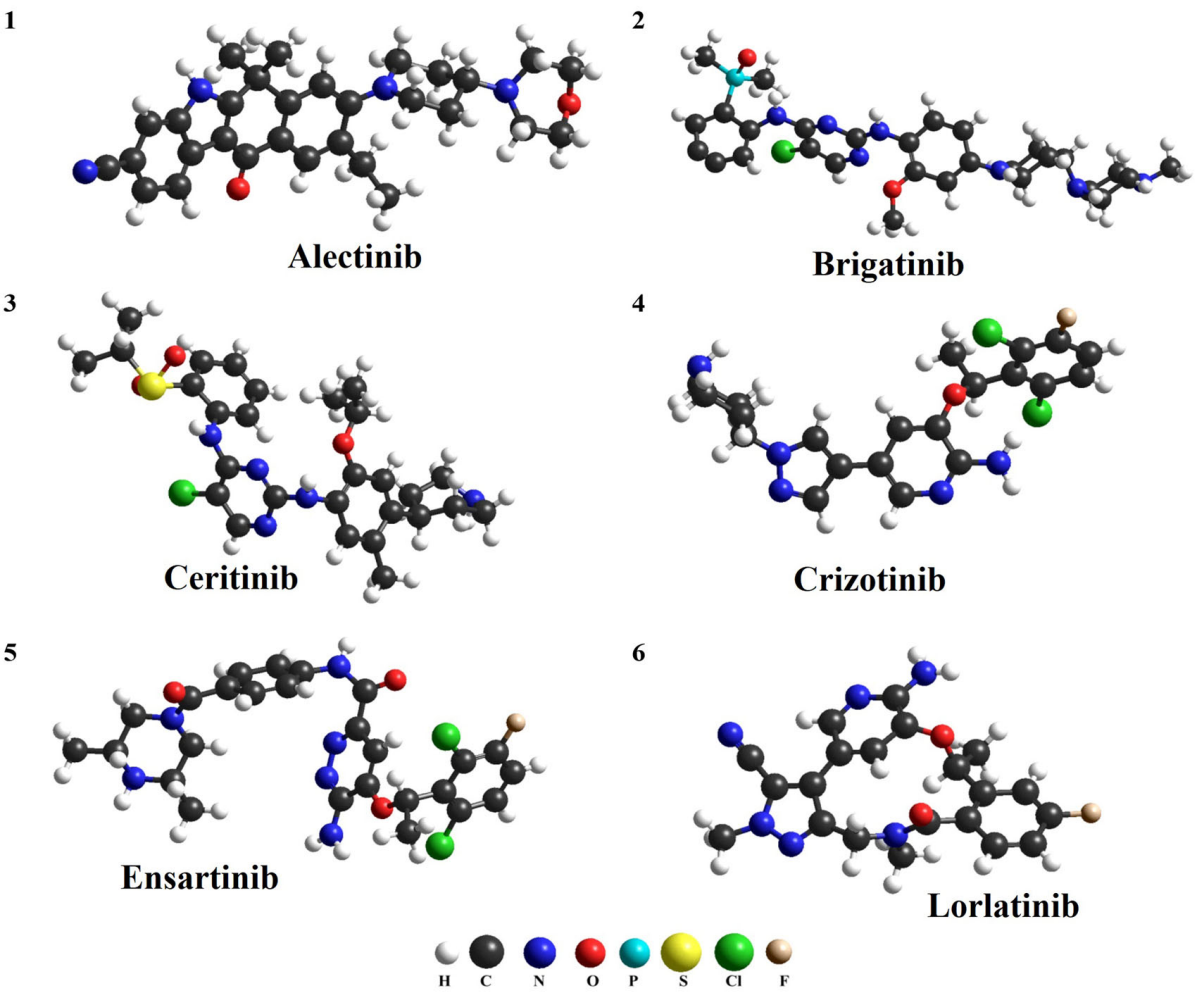
Structure and physicochemical properties of ALK inhibitors (H: hydrogen; C: carbon; N: nitrogen; O: oxygen; P: phosphorus; S: sulfur; Cl: chlorine; F: fluorine).

One of the most common problems with ALK inhibitors (ALKi) is multi-drug resistance. The mechanism of resistance development is yet not fully understood. It can be generally divided into ALK-dependent and ALK-independent factors. The first type of resistance factors includes mutations in the *ALK* gene, causing structural changes and interfering with the binding of the drug. Approximately 30%–40% of all currently known resistance mechanisms belong to the kinase domain mutations [24] ALK-independent factors include, but not limited to, drug efflux pump, driven by the family of P-glycoproteins [25]. P-gp is an ATP-driven transporter for hydrophobic drugs and it is responsible for multidrug resistance, due to significantly reducing the concentration of ALK inhibitors in the central nervous system [26]. It has been found that poor BBB penetration is the main reason of crizotinib failure in brain metastasis prevention and treatment [4]. It is not clear which features of the ALK inhibitors have a crucial impact on crossing BBB. The strategies of increasing the CNS concentration of ALK inhibitors include the development of new substances, which are not transported *via* P-gp or modifying already existing structures by the introduction of lipophilic moiety [27]. The structural representation of selected ALK inhibitors with selected physiochemical properties are shown in Figure 1.

The pharmacodynamic and pharmacokinetic properties of drugs depend on their chemical structure and physicochemical properties. There are certain molecular parameters of compounds that determine their absorption, distribution, metabolism and excretion profile. The values of these parameters for drugs administered orally are characterized by the so-called Lipinski’s rule of five. According to it, a molecule with the desired pharmacokinetic properties should be characterized by the following parameters: molecular mass less than 500 daltons, no more than 5 hydrogen bond donors, no more than 10 hydrogen bond acceptors, an octanol-water partition coefficient (log P) that does not exceed 5. A slight breach of this principle (with no more than one derogation) does not imply that the compound cannot be an effective drug, but two derogations dramatically reduce the chance of being effective [28]. As presented in Figure 1 only crizotinib and lorlatinib fulfill all the criteria of rule of five, brigatinib, ceritinib and ensartinib have molecular weights above 500 Da, and the values of lipophilicity parameter (LogP) of alectinib and ceritinib exceed the limit of the rule. On the other hand, all compounds presented in Figure 1 satisfy Veber’s rule, who predicted that molecules with a polar surface of less than 140 Å2 and 10 or less rotary bonds will be characterized by good bioavailability [29]. Due to the fact that crizotinib meets Lipiński’s rule (and modern inhibitors do not necessarily) and that all the mentioned compounds fulfill Veber’s rule, it is difficult to find a direct link between the basic physicochemical parameters of these drugs and the effectiveness of treatment.

Crizotinib is an orally available first generation ALK inhibitor and signal transduction c-ros oncogene 1 (ROS1) kinase. Its bioavailability after a single dose orally-administered is calculated on approximately 43%, the median time to achieve its peak concentration (*t*_max_) is 4–6 h and an apparent terminal half-life (*t*_1/2_) is 42 h. Crizotinib is mainly excreted in the feces and to a lesser extend in the urine. It is found that crizotinib is a substrate for P-glycoprotein therefore penetration of BBB by the crizotinib is weak. The cerebrospinal fluid/plasma (CSF/plasma) ratio of crizotinib is calculated on only 0.003. These findings are consistent with the ineffectiveness of crizotinib therapy in patients with brain metastases [30–32].

Ceritinib is a second-generation ALK inhibitor with more potent affinity and activity against ALK than its forerunner, crizotinib. Ceritinib is also a P-gp substrate, which leads to limited BBB penetration, but CSF/plasma ratio is higher (0.13–0.35) than in the case of crizotinib use. Metabolism of ceritinib is mediated mainly by P450 hepatic cytochrome CYP3A4. 11 metabolites were found, yet the form with the most contribution in the plasma (82%) was unchanged ceritinib [31,33,34].

Alectinib, an orally-administered second-generation ALK inhibitor, is characterized by good penetration through BBB and it can achieve high concentrations in the CSF. It is recognized, that alectinib is not a substrate to P-gp, which can explain its effectiveness in brain metastases prevention and treatment. The CSF/plasma ratio of alectinib was estimated on 0.75 [31,35]. Alectinib is metabolized primarily by CYP3A4 and it may be converted to a clinically relevant active metabolite known as M4. Both alectinib and M4 are not transported by P-gp [36].

Brigatinib is an oral ALK inhibitor which belongs to the second generation of ALKi. Peak concentration after an orally-administered single dose is achieved earlier (1–4 h) than in the case of crizotinib use. Brigatinib is also metabolized mainly by CYP3A4, but it excreted in a greater significant amount in urine than crizotinib, ceritinib and alectinib, which indicates an alternative pathway of brigatinib elimination. Its active metabolite (AP26123) is 3-fold less potent in ALK inhibition than the parent compound. The contribution of AP26123 in the plasma is merely 3.5% [31,37]. Although brigatinib was described as a more effective drug than crizotinib in CNS progression, its CSF/plasma ratio is estimated on only 0.31 [35]. What is more, brigatinib is found to be a substrate to the P-gp efflux pump, and its efficacy in brain metastases prophylaxis and treatment may stem from pharmacodynamic properties.

Lorlatinib is a third-generation of ALK inhibitor. It is estimated that lorlatinib reaches its peak concentration in approximately 1–2 h and bioavailability is calculated on circa 81% after oral administration. Lorlatinib is metabolized primarily by CYP3A4 and excreted mainly with feces. Among the group of ALKi, only lortatinib has a macrocyclic structure (see Figure 1), and, as it is suggested, this feature is beneficial for gaining high concentrations in CNS. Lorlatinib is a weak substrate for P-gp [38,39]. However, the mechanism of lorlatinib penetration of BBB is complex and yet not fully understood [40]. The CSF/plasma ratio of lorlatinib is high, and it is estimated on 0.75 [35].

Ensartinib is a novel molecule, approximately 10-fold more potent than crizotinib at inhibiting cancer cell lines with *ALK* gene rearrangement. The drug is still in phase 3. clinical trials (eXalt3) and a very limited amount of data is available [41]. The preliminary results from clinical trials phase 1/2 suggest that ensartinib seems to be a promising drug in the treatment of NSCLC patients with *ALK* gene rearrangement and CNS metastases [42,43]. Some selected pharmacokinetic parameters of ALK inhibitors including ensartinib are given in Table 2.

**Table 2.** Selected properties of ALK inhibitors.

Parameter/drug	Crizotinib	Ceritinib	Alectinib	Brigatinib	Lorlatinib	Ensartinib
Year of approval (FDA)	2011	2014	2015	2017	2018	Phase 3. clinical trial
Generation	I	II	II	II	III	II
Route of administration	Oral	Oral	Oral	Oral	Oral	Oral
Available dosage	200 mg; 250 mg	150 mg	150 mg	30 mg; 60 mg; 180 mg	25 mg; 100 mg	225 mg
Approved dosage	Twice daily	Once daily	Twice daily	Once daily	Once daily	Once daily
Bioavailability (oral)	43%	25%	36,9%	ND	81%	ND
*t*_max_	4–6h	4–6 h	4–6 h	1–4 h	1–2 h	3–4 h
*t*_1/2_	42 h	31–41 h	32 h	24 h	15 h	21–30 h
P-gp affinity	Substrate	Substrate	No affinity	Substrate	Substrate	Substrate
CSF/plasma ratio	0.003	0.13–0.35*	0.75	0.31	0.75	0.016**
References	Ceddia and Codacci-Pisanelli (2021) [35] Hirota et al. (2019) [31] XALKORI^®^ SmPC (2016) [32]	Chow et al. (2022) [33] Hirota et al. (2019) [31] ZYKADIA^®^ SmPC (2017) [34]	Ceddia and Codacci-Pisanelli (2021) [35] Hirota et al. (2019) [31] ALECENSA^®^ SmPC (2017) [36]	Ceddia and Codacci-Pisanelli (2021) [35] ALUNBRIG^®^ SmPC (2018) [37]	Ceddia and Codacci-Pisanelli (2021) [35] LORVIQUA^®^ SmPC (2021) [39]	Zhao et al. (2020) [43] Horn et al. (2018) [42]

ND: no data found; *t*_max_: median time to achieve peak concentration; *t*_1/2_: apparent terminal half-life; CSF: cerebrospinal fluid; FDA: Food and Drug Administration; SmPC: Summary of Product Characteristics.

* Range of calculated ratio in studied group.

** Data from I phase clinical trial.

## 5. Effectiveness of first generation of ALK inhibitors in NSCLC CNS metastases treatment

In the PROFILE 1014 study, which documented the superiority of crizotinib over chemotherapy in the first-line treatment of ALK-positive NSCLC patients, the intracranial activity of the drug was also analysed prospectively. The crizotinib-treated group had a higher intracranial overall response rate (ORR), both at 12 and 24 weeks of follow-up −85% vs. 45% and 56% vs. 25%, respectively. There was a trend towards longer time to intracranial progression in patients both with and without baseline CNS secondary lesions. The differences were statistically insignificant [44]. The limited intracranial efficacy of crizotinib was also demonstrated in the studies preceding the registration of second and third-generation ALK inhibitors, which are discussed later in this publication.

## 6. Effectiveness of second generation of ALK inhibitors in NSCLC CNS metastases treatment

### 6.1. Alectinib

In ALEX – phase 3. clinical trials, CNS imaging was mandatory for all patients eligible for the treatment. Patients with disease progression in the form of asymptomatic CNS metastases were allowed – after local treatment (radiotherapy) – to continue systemic treatment until systemic disease progression, symptomatic CNS progression, or both [45]. In the ALEX trial, radiological assessment (computer tomography – CT or magnetic resonance – MR) was conducted every 8 weeks. Secondary endpoints of the study included progression-free survival (PFS) and ORR of CNS lesions as assessed by RECIST 1.1 (Response Evaluation Criteria In Solid Tumors) classification criteria.

Of the 303 patients enrolled in the study, the presence of CNS lesions was confirmed in 122 patients (58 patients received crizotinib, 64 patients – alectinib) [46]. In 43 patients, the lesions were defined as measurable, and 46 patients had previously undergone CNS irradiation.

The superiority of alectinib over crizotinib in terms of ORR was demonstrated, irrespective of prior local treatment. Intracranial treatment response was noted in 85.7% vs. 71.4% of patients after irradiation and in 78.6% and 40% of patients without local treatment, respectively. The duration of response (including measurable and non-measurable changes) was longer in alectinib-treated patients, both after prior radiotherapy (median not reached for alectinib, while it was 11.1 months for crizotinib) and without local treatment (median not reached for alectinib, while it was 3.7 months for crizotinib) [46]. Subgroup analysis showed that CNS progression at 12 months occurred in 12% of patients in the alectinib group compared with 45% of patients in the crizotinib group (HR = 0.16; *p* < .001). Median PFS in patients with primary CNS metastases and patients without CNS metastases for alectinib and crizotinib was 25.4 vs. 7.4 months and 38.6 vs. 14.8 months, respectively [47].

Of the 207 patients qualified for treatment in the J-ALEX trial, the presence of CNS metastases was found in 43 patients (29 received crizotinib, 14 – alectinib). In some cases, prior brain irradiation was performed (among patients treated with alectinib − 51.6%, among patients treated with crizotinib − 37.5%) [48]. In the entire analysed population, the percentage of patients with progression of CNS lesions after 12 months of follow-up was lower in patients treated with alectinib than in those treated with crizotinib (5.9% vs. 16.8%, respectively). Differences in time to CNS progression were noted both in patients with metastatic lesions present at baseline (HR = 0.51; *p* = .025) and in patients without CNS lesions (HR = 0.19; *p* = .0004) [48].

In the ALESIA study, brain metastases were initially found in 67 of 187 patients. Prior irradiation was performed in 6% and 8% of patients enrolled for alectinib or crizotinib therapy, respectively [49]. There was a significant reduction in the risk of CNS disease progression associated with alectinib (HR = 0.14). The percentage of patients with confirmed CNS progression after 12 months was 7.3% for alectinib and 35.5% for crizotinib [49]. The superiority of alectinib in terms of intracranial ORR was also demonstrated, with the percentages of patients (combined analysis of measurable and non-measurable lesions) with a response to the treatment being 73% and 22%, respectively [49]. Among patients in whom only measurable changes were assessed, an objective response was observed in 2 patients (29% of 7 patients) treated with crizotinib and in 16 patients (94% of 17 patients) treated with alectinib.

The activity of alectinib was also documented in patients after the failure of treatment with crizotinib and chemotherapy (ALUR study). Median PFS was significantly longer for alectinib (9.6 vs. 1.4 months; HR = 0.15; *p* <.001); intracranial ORR (icORR) was 54.2% in alectinib-treated patients and 0% in chemotherapy-treated patients. The incidence of CNS progression at 6 months was 11% and 48%, respectively [50].

### 6.2. Brigatinib

A pooled analysis of patients who were treated with brigatinib in the phase 1/2 clinical trial and the phase 2 ALTA trial has been published [51]. The phase 1/2 clinical trial enrolled 79 patients with *ALK* rearrangement. Two dosing regimens of brigatinib were used in the ALTA trial. In arm A, 112 patients received 90 mg of brigatinib once daily. However, in arm B, 110 patients were treated with brigatinib in the dose of 180 mg once daily following a seven-day lead-in of 90 mg once daily [52]. The majority of patients enrolled to phase 1/2 and ALTA clinical trials had brain metastases at baseline. In these clinical trials, brain metastases were manifested in 63% and 69% of patients, respectively. In the 1/2 phase clinical trial, 54% of them received brain radiotherapy prior brigatinib therapy. Whereas, 74% of patients in the ALTA trial had received prior chemotherapy. 53% of patients had confirmed intracranial response in the phase 1/2 clinical trial. In arm A and in arm B of the ALTA trial, 42% and 67% of patients had an objective intracranial response. Prior radiotherapy had no effect on the chance of the occurrence of intracranial response. Median intracranial progression-free survival (icPFS) was 14.6 months (95% CI: 12.7–36.8 months) in patients from the phase 1/2 clinical trial, 15.6 months (95% CI: 9.0–18.3 months) in patients from arm A and 12.8 months in patients from arm B of ALTA trial [51,52].

The ALTA-1L trial compared the efficacy and safety of brigatinib and crizotinib in treatment-naïve patients with *ALK* gene rearrangement. 81 patients qualified to first-line therapy had brain metastases at baseline (40 treated with brigatinib and 41 treated with crizotinib). In this group, The intracranial response was confirmed in 78% of patients treated with brigatinib and in 26% of patients who received crizotinib [53]. An updated analysis of the ALTA-1L trial results confirmed the higher intracranial efficacy of brigatinib than crizotinib [54]. 31% of patients with brain metastases at baseline and treated with brigatinib had no progression after 3 years of observation. However, only 9% of such patients were in the group receiving crizotinib (HR = 0.29; 95% CI: 0.17–0.51, *p* < .0001). After 4 years of follow-up, 22% of patients treated with brigatinib were without intracranial progression. In contrast, there was no patient without intracranial progression in the patients who received crizotinib. Median icPFS was 24.0 months (95% CI: 12.9–30.8) and 5.5 months (95% CI: 3.7–7.5 months), respectively (HR = 0.29; 95% CI: 0.17–0.51; *p* < .0001). In patients with brain metastases at baseline, 74% of patients treated with brigatinib and 55% of patients receiving crizotinib had survival longer than three years, and the hazard ratio for death was 0.43 (95% CI: 0.21–0.89, *p* = .020). The surival benefit was greater in the brigatinib group compared to the crizotinib group, especially in the patients not undergoing brain radiotherapy (HR = 0.25, 95% CI: 0.08–0.75, *p* = .008) than in patients with previous brain radiotherapy (HR = 0.76, 95% CI: 0.27–2.12, *p* = .637). What is extremely important, brigatinib compared to criztotinib significantly improved the patients’ quality of life (QoL).The median time to worsening of QoL in patients treated with brigatinib was 26.7 months. However, it was only 8.3 months in patients who received crizotinib. Brigatinib significantly delayed the time to worsening of emotional and social functioning as well as it prolonged the time until the appearance of fatigue, nausea, vomiting, appetite loss, and constipation. This concerned both patients with and without brain metastases [54].

### 6.3. Ceritinib

The ASCENT-1 study was designed to demonstrate the efficacy and safety of ceritinib in patients with advanced NSCLC with *ALK* gene rearrangement after the failure of standard therapy (including prior crizotinib therapy). This study recruited 246 patients, 124 of whom had brain metastases [52]. In 94 patients, brain metastases were confirmed retrospectively used at least one post-baseline MRI or CT assessment. In this group, the intracranial disease control rate was 79% in crizotinib naïve patients (15 out of 19 patients) and 65% in patients who previously received crizotinib (49 out of 75 patients). In the group of 11 patients with measurable brain lesions and no previous CNS radiotherapy, six individuals achieved a partial intracranial response [52].

In the ASCEND-4 trial, the value of ceritinib was evaluated in patients who had not received systemic treatment [55]. The trial enrolled 187 patients and 31% of patients had CNS metastatic lesions at the baseline (the majority – about 80% – had received radiotherapy). In patients treated with ceritinib, the icORR was 72.7% (HR = 0.58) 27.3% patients who previously received chemotherapy showed intracranial resposne to ceritinib. The benefit of ceritinib treatment, as measured by overall progression-free survival, was achieved by both patients with CNS metastases and patients without secondary lesions in this location. However, it was lower for patients with CNS metastases. In the group of ceritinib-treated patients, the median PFS for patients with and without metastases was 10.7 vs. 26.3 months. In the group of patients who received chemotherapy, the mPFS was 6.7 and 8.3 months, respectively. In patients with CNS metastases and without them, ceritinib was more effective than chemotherapy (HR = 0.70; 95% CI: 0.44–1.12 and HR = 0.48; 95% CI: 0.33–0.69, respectively) [55]. Detailed data on intracranial PFS were not provided.

## 7. Third generation ALK inhibitors effectiveness in NSCLC CNS metastases treatment

### 7.1. Lorlatinib

Lorlatinib is a third-generation ALKi, active against cancer cells with rearrangements in the *ALK* and *ROS1* genes. In preclinical studies and in the initial phases of clinical trials, it had a high intracranial activity with icORR of up to 87% [56].

Results of a phase II study (NCT01970865) including a group of 198 patients with ≥1 prior ALKi treatment have been presented [57]. In a group of 59 patients previously treated with crizotinib, the cumulative incidence rate (CIR) of CNS progression at 12 months was 22% in patients with baseline CNS metastases (*n* = 37) and 9% in patients without CNS metastases (*n* = 22). In patients who received ≥1 prior second-generation ALKi treatment (*n* = 139), the CIR of CNS progression at 12 months was 43% and 9%, respectively [57].

In the population analysed in the CROWN trial which compared lorlatinib with crizotinib in previously untreated patients, the intracranial response was prospectively observed in a group of 78 patients with primary measurable and non-measurable brain lesions [58]. CNS magnetic resonance imaging was mandatory for all patients in the screening phase of the study. Secondary brain lesions were found in 26% of patients and 6% of patients had received prior brain irradiation. Objective intracranial responses were noted in 82% and 23% of patients, including complete responses (CR) in 71% and 8% of patients treated with lorlatinib or crizotinib, respectively. Among 30 patients with measurable lesions, 82% in the lorlatinib group and 23% in the crizotinib group achieved an intracranial response, and 71% and 8% achieved complete remission. There was a significant reduction in the risk of intracranial lesion progression (HR = 0.07; 95% CI: 0.03–0.17). The cumulative incidence of intracranial progression as first treatment failure was significantly lower in the lorlatinib treated group than in patients who received crizotinib. After 12 months, the cumulative risk of CNS progression was 2.8% in patients treated with lorlatinib compared with 33% in patients in the control arm (HR = 0.06; 95% CI: 0.02–0.18) [58].

## 8. Ensartinib effectiveness in NSCLC CNS metastases treatment

Preliminary phase 1/2 clinical trials proved ensartinib activity in the treatment of central nervous system metastases [42]. In a group of 14 patients with CNS metastases at baseline, 2 achieved icCR (both had no prior irradiation) and 7 achieved icPR (5 had no prior irradiation and 2 had prior irradiation). The ORR was 64.3% [42].

The phase III EXALT trial compared ensartinib with crizotinib in a group of 290 patients previously untreated with ALKi (1. line of chemotherapy was allowed) [59]. Median PFS was significantly longer in patients treated with ensartinib than in patients received crizotinib (25.8 vs. 12.7 months; HR = 0.51; 95% CI: 0.35–0.72; *p* < .001). Metastatic lesions at baseline were found in 33% of ensartinib treated patients and in 39% of crizotinib-treated patients. In both subgroups, prior radiotherapy was used in approximately 5% of patients. Median PFS for patients with CNS metastases received ensartinib and crizotinib were 11.8 months vs. 7.5 months, respectively (HR = 0.55; 95% CI: 0.30–1.01; *p* = .05). Eleven patients receiving ensartinib and 19 patients receiving crizotinib had measurable CNS metastases. The icORR in this group was observed in 63.6% and 21.1% of patients, respectively. In the group of patients without brain metastases at baseline, ensartinib significantly reduced the risk of CNS metastases. After 12 months, metastases were observed in 4.2% patients treated with ensartinib and 23.9% patients treated with crizotinib (HR = 0.32; 95% CI: 0.16–0.63; *p* = .001) [59] (Table 3).

**Table 3.** The risk reduction of CNS disease progression associated with the use of ALK inhibitors.

Clinical trials	Treatment	micPFS in months (for patients with CNS metastases)	HR	2 months icPFS in % (for patients with CNS metastases)	HR	CIR at 12 months (%)	HR
ALEX Gadgeel et al. [46]	alectinib vs. crozotinib	ND	ND	ND	ND	9.4 vs. 41.4	ND
ALTA-1 Cmidge et al. [53,54]	brigatinib vs. crizotinib	24 vs. 5.5	0.29	74 vs. 67 (24 months)	0.78 (NCS)	ND	ND
ASCEND-4 Soria et al. [55]	ceritinib vs. chemotherapy	ND	ND	ND	ND	ND	ND
EXALT Horn et al. [59]	ensartinib vs. crizotinib	11.8 vs. 7.5	0.55	ND	ND	4.2 vs. 23.9	0.32
CROWN Shaw et al. [58]	lorlatinib vs. crizotinib	NE vs. 16.6*	0.07	ND	ND	3 vs. 33	0.06

micPFS: median of intracranial progression free surivial; ND: no data; HR: hazard ratio; CIR: cumulative incidence rate.

* Intracranial time to progression.

## 10. Conclusions

CNS metastases are very common in NSCLC patients with *ALK* gene rearrangement. Previous methods of brain metastases treatment have been insufficient (radiotherapy, chemotherapy). The discovery of ALK inhibitors has revolutionized the management of patients with CNS metastases. The first-generation inhibitor, crizotinib, was characterized by low penetration by the BBB. However, second and third-generation inhibitors show significant efficacy in the treatment of CNS metastases. In many patients with asymptomatic brain metastases at baseline, it is possible to resign from brain irradiation and start treatment with ALKi therapy. Moreover, a new generation of ALK inhibitors reduce the risk of metastases in patients who did not have metastases at the beginning of the disease. The problem of resistance to ALK inhibitors continues. However, progression-free survival (including intracranial PFS) and overall survival of NSCLC patients with ALK rearrangement significantly prolonged by the use of new drugs such as lorlatinib in first line therapy.

## Data Availability

The data that support the findings of this study are available from the corresponding author, [M.G.], upon reasonable request.

## References

[1] Li M, Hou X, Zhou C, et al. Prevalence and clinical impact of concomitant mutations in anaplastic lymphoma kinase rearrangement advanced non-small-cell lung cancer (Guangdong Association of Thoracic Oncology Study 1055). Front Oncol. 2020;10:1216. 32974126 10.3389/fonc.2020.01216 PMC7471725

[2] Rosenbaum JN, Bloom R, Forys JT, et al. Genomic heterogeneity of ALK fusion breakpoints in non-small-cell lung cancer. Mod Pathol. 2018;31(5):791–808. 29327716 10.1038/modpathol.2017.181

[3] Petrelli F, Lazzari C, Ardito R, et al. Efficacy of ALK inhibitors on NSCLC brain metastases: a systematic review and pooled analysis of 21 studies. PLOS One. 2018;13(7):e0201425. 10.1371/journal.pone.0201425 PMC6063430 30052658

[4] Zhang I, Zaorsky NG, Palmer JD, et al. Targeting brain metastases in ALK-rearranged non-small-cell lung cancer. Lancet Oncol. 2015;16(13):e510–e521. 26433824 10.1016/S1470-2045(15)00013-3

[5] Ettinger D, Wood D, Aisner D, et al. Non-Small Cell Lung Cancer, Version 3. 2022. J Natl Compr Canc Netw. 2022;20:497-530. 10.6004/jnccn.2022.0025 35545176

[6] Abbott NJ, Patabendige AAK, Dolman DEM, et al. Structure and function of the blood-brain barrier. Neurobiol Dis. 2010;37(1):13–25. 19664713 10.1016/j.nbd.2009.07.030

[7] Daneman R, Prat A. The blood-brain barrier. Cold Spring Harb Perspect Biol. 2015;7(1):a020412. 10.1101/cshperspect.a020412 PMC4292164 25561720

[8] Liebner S, Dijkhuizen RM, Reiss Y, et al. Functional morphology of the blood-brain barrier in health and disease. Acta Neuropathol. 2018;135(3):311–336. 29411111 10.1007/s00401-018-1815-1 PMC6781630

[9] Keaney J, Campbell M. The dynamic blood-brain barrier. FEBS J. 2015;282(21):4067–4079. 26277326 10.1111/febs.13412

[10] Benz F, Liebner S. Structure and function of the blood-brain barrier (BBB). Handb Exp Pharmacol. 2022;273:3–31. 33249527 10.1007/164_2020_404

[11] Mahringer A, Fricker G. ABC transporters at the blood-brain barrier. Expert Opin Drug Metab Toxicol. 2016;12(5):499–508. 26998936 10.1517/17425255.2016.1168804

[12] Miller DS. Regulation of ABC transporters blood-brain barrier: the good, the bad, and the ugly. Adv Cancer Res. 2015;125:43–70. 25640266 10.1016/bs.acr.2014.10.002

[13] Pardridge WM. Drug transport across the blood-brain barrier. J Cereb Blood Flow Metab. 2012;32(11):1959–1972. 22929442 10.1038/jcbfm.2012.126 PMC3494002

[14] Leslie EM, Deeley RG, Cole SPC. Multidrug resistance proteins: role of P-glycoprotein, MRP1, MRP2, and BCRP (ABCG2) in tissue defense. Toxicol Appl Pharmacol. 2005;204(3):216–237. 15845415 10.1016/j.taap.2004.10.012

[15] Schinkel AH. P-Glycoprotein, a gatekeeper in the blood-brain barrier. Adv Drug Deliv Rev. 1999;36(2-3):179–194. 10837715 10.1016/s0169-409x(98)00085-4

[16] Pardridge WM. The blood-brain barrier: bottleneck in brain drug development. NeuroRx. 2005;2(1):3–14. 15717053 10.1602/neurorx.2.1.3 PMC539316

[17] Pardridge WM. Blood-brain barrier delivery. Drug Discov Today. 2007;12(1–2):54–61. 17198973 10.1016/j.drudis.2006.10.013

[18] Robey RW, Pluchino KM, Hall MD, et al. Revisiting the role of ABC transporters in multidrug-resistant cancer. Nat Rev Cancer. 2018;18(7):452–464. 29643473 10.1038/s41568-018-0005-8 PMC6622180

[19] Doyle LA, Ross DD. Multidrug resistance mediated by the breast cancer resistance protein BCRP (ABCG2). Oncogene. 2003;22(47):7340–7358. 14576842 10.1038/sj.onc.1206938

[20] Gil-Martins E, Barbosa DJ, Silva V, et al. Dysfunction of ABC transporters at the blood-brain barrier: role in neurological disorders. Pharmacol Ther. 2020;213:107554. 32320731 10.1016/j.pharmthera.2020.107554

[21] Wrona A. Management of CNS disease in ALK-positive non-small cell lung cancer: is whole brain radiotherapy still needed? Cancer Radiother. 2019;23(5):432–438. 31331844 10.1016/j.canrad.2019.03.009

[22] Zweig JR, Neal JW. Infiltrating the blood-brain barrier in ALK-positive lung cancer. J Clin Oncol. 2018;36(26):2677–2679. 29939839 10.1200/JCO.2018.78.8554

[23] Tatineni V, O'Shea PJ, Rauf Y, et al. Outcomes of first, second, and third-generation anaplastic lymphoma kinase (ALK) inhibitors in non-small cell lung cancer brain metastases (NSCLCBM). J Clin Oncol. 2021;39(15 Suppl):2034–2034.

[24] Pan Y, Deng C, Qiu Z, et al. The resistance mechanisms and treatment strategies for ALK-rearranged non-small cell lung cancer. Front Oncol. 2021;11:713530. 34660278 10.3389/fonc.2021.713530 PMC8517331

[25] Elsayed M, Christopoulos P. Therapeutic sequencing in ALK + NSCLC. Pharmaceuticals. 2021;14(2):80–18. 33494549 10.3390/ph14020080 PMC7912146

[26] Váradi A, Szakács G, Bakos É, et al. P glycoprotein and the mechanism of multidrug resistance. Novartis Found Symp. 2002;243:54–68. 11990782

[27] Radaram B, Pisaneschi F, Rao Y, et al. Novel derivatives of anaplastic lymphoma kinase inhibitors: synthesis, radiolabeling, and preliminary biological studies of fluoroethyl analogues of crizotinib, alectinib, and ceritinib. Eur J Med Chem. 2019;182:111571. 31425908 10.1016/j.ejmech.2019.111571 PMC7193943

[28] Lipinski CA, Lombardo F, Dominy BW, et al. Experimental and computational approaches to estimate solubility and permeability in drug discovery and development settings. Adv Drug Deliv Rev. 2001;46(1–3):3–26. 11259830 10.1016/s0169-409x(00)00129-0

[29] Veber DF, Johnson SR, Cheng HY, et al. Molecular properties that influence the oral bioavailability of drug candidates. J Med Chem. 2002;45(12):2615–2623. 12036371 10.1021/jm020017n

[30] Okimoto T, Tsubata Y, Hotta T, et al. A low crizotinib concentration in the cerebrospinal fluid causes ineffective treatment of anaplastic lymphoma kinase-positive non-small cell lung cancer with carcinomatous meningitis. Intern Med. 2019;58(5):703–705. 30333394 10.2169/internalmedicine.1072-18 PMC6443566

[31] Hirota T, Muraki S, Ieiri I. Clinical pharmacokinetics of anaplastic lymphoma kinase inhibitors in non-small-cell lung cancer. Clin Pharmacokinet. 2019;58(4):403–420. 29915924 10.1007/s40262-018-0689-7

[32] European Medicines Agency. Xalkori - Summary of Product Characteristics (SmPC), 2016 [online]. Available at: https://www.ema.europa.eu/en/documents/product-information/xalkori-epar-product-information_en.pdf. Accessed 10 December 2022.

[33] Chow LQM, Barlesi F, Bertino EM, et al. ASCEND-7: efficacy and safety of ceritinib treatment in patients with ALK-Positive Non-Small cell lung cancer metastatic to the brain and/or leptomeninges. Clin Cancer Res. 2022;28(12):2506–2516. 35091443 10.1158/1078-0432.CCR-21-1838

[34] European Medicines Agency. Zykadia - Summary of Product Characteristics (SmPC), 2017 [online]. Available at: https://www.ema.europa.eu/en/documents/product-information/zykadia-epar-product-information_en.pdf. Accessed 10 December 2022.

[35] Ceddia S, Codacci-Pisanelli G. Treatment of brain metastases in ALK-Positive Non-Small cell lung cancer. Crit Rev Oncol Hematol. 2021;165:103400. 34147645 10.1016/j.critrevonc.2021.103400

[36] European Medicines Agency. Alecensa - Summary of Product Characteristics (SmPC), 2017 [online]. Available at: https://www.ema.europa.eu/en/documents/product-information/alecensa-epar-product-information_en.pdf. Accessed 10 December 2022.

[37] European Medicines Agency. Alunbrig - Summary of Product Characteristics (SmPC), 2018 [online]. Available at: https://www.ema.europa.eu/en/documents/product-information/alunbrig-epar-product-information_en.pdf. Accessed 10 December 2022.

[38] Akamine T, Toyokawa G, Tagawa T, et al. Spotlight on lorlatinib and its potential in the treatment of NSCLC: the evidence to date. Onco Targets Ther. 2018;11:5093–5101. 30174447 10.2147/OTT.S165511 PMC6110295

[39] European Medicines Agency. Lorviqua - Summary of Product Characteristics (SmPC), 2021 [online]. Available at: https://www.ema.europa.eu/en/documents/product-information/lorviqua-epar-product-information_en.pdf. Accessed 10 December 2022.

[40] Chen W, Jin D, Shi Y, et al. The underlying mechanisms of lorlatinib penetration across the Blood-Brain barrier and the distribution characteristics of lorlatinib in the brain. Cancer Med. 2020;9(12):4350–4359. 32347012 10.1002/cam4.3061 PMC7300403

[41] Fukui T, Tachihara M, Nagano T, et al. Review of therapeutic strategies for anaplastic lymphoma kinase-rearranged non-small cell lung cancer. Cancers. 2022;14(5):1184. 10.3390/cancers14051184 PMC8909087 35267492

[42] Horn L, Infante JR, Reckamp KL, et al. Ensartinib (X-396) in ALK-positive non-small cell lung cancer: results from a first-in-human phase I/II, multicenter study. Clin Cancer Res. 2018;24(12):2771–2779. 29563138 10.1158/1078-0432.CCR-17-2398 PMC6004248

[43] Zhao H, Ma Y, Zhang Y, et al. Abstract 579: ensartinib (X-396), a novel ALK TKI, in chinese ALK-positive non-small cell lung cancer: a phase I, dose-escalation and expansion study. Cancer Res. 2020;80(16_Suppl):579–579.

[44] Solomon BJ, Cappuzzo F, Felip E, et al. Intracranial efficacy of crizotinib versus chemotherapy in patients with advanced ALK-positive non-small-cell lung cancer: results from PROFILE 1014. J Clin Oncol. 2016;34(24):2858–2865. 27022118 10.1200/JCO.2015.63.5888

[45] Camidge DR, Dziadziuszko R, Peters S, et al. Updated efficacy and safety data and impact of the EML4-ALK fusion variant on the efficacy of alectinib in untreated ALK-positive advanced non-small cell lung cancer in the global phase III ALEX study. J Thorac Oncol. 2019;14(7):1233–1243. 30902613 10.1016/j.jtho.2019.03.007

[46] Gadgeel S, Peters S, Mok T, et al. Alectinib versus crizotinib in treatment-naive anaplastic lymphoma kinase-positive (ALK+) non-small-cell lung cancer: CNS efficacy results from the ALEX study. Ann Oncol. 2018;29(11):2214–2222. 30215676 10.1093/annonc/mdy405 PMC6290889

[47] Mok T, Camidge DR, Gadgeel SM, et al. Updated overall survival and final progression-free survival data for patients with treatment-naive advanced ALK-positive non-small-cell lung cancer in the ALEX study. Ann Oncol. 2020;31(8):1056–1064. 32418886 10.1016/j.annonc.2020.04.478

[48] Nishio M, Nakagawa K, Mitsudomi T, et al. Analysis of central nervous system efficacy in the J-ALEX study of alectinib versus crizotinib in ALK-Positive non-small-cell lung cancer. Lung Cancer. 2018;121:37–40. 29858024 10.1016/j.lungcan.2018.04.015

[49] Zhou C, Kim SW, Reungwetwattana T, et al. Alectinib versus crizotinib in untreated asian patients with anaplastic lymphoma kinase-positive non-small-cell lung cancer (ALESIA): A randomised phase 3 study. Lancet Respir Med. 2019;7(5):437–446. 30981696 10.1016/S2213-2600(19)30053-0

[50] Novello S, Mazières J, Oh IJ, et al. Alectinib versus chemotherapy in crizotinib-pretreated anaplastic lymphoma kinase (ALK)-positive non-small-cell lung cancer: results from the phase III ALUR study. Ann Oncol. 2018;29(6):1409–1416. 29668860 10.1093/annonc/mdy121 PMC6005013

[51] Ross Camidge D, Kim DW, Tiseo M, et al. Exploratory analysis of brigatinib activity in patients with anaplastic lymphoma kinase-positive non-small-cell lung cancer and brain metastases in two clinical trials. J Clin Oncol. 2018;36(26):2693–2701. 29768119 10.1200/JCO.2017.77.5841

[52] Kim DW, Mehra R, Tan DSW, et al. Activity and safety of ceritinib in patients with ALK-rearranged non-small-cell lung cancer (ASCEND-1): updated results from the multicentre, open-label, phase 1 trial. Lancet Oncol. 2016;17(4):452–463. 26973324 10.1016/S1470-2045(15)00614-2 PMC5063047

[53] Camidge DR, Kim HR, Ahn MJ, et al. Brigatinib versus crizotinib in advanced ALK inhibitor-naive ALK-Positive non-small cell lung cancer: second interim analysis of the phase III ALTA-1L trial. J Clin Oncol. 2020;38(31):3592–3603. 32780660 10.1200/JCO.20.00505 PMC7605398

[54] Camidge DR, Kim HR, Ahn MJ, et al. Brigatinib versus crizotinib in ALK inhibitor-naive advanced ALK-positive NSCLC: final results of phase 3 ALTA-1L trial. J Thorac Oncol. 2021;16(12):2091–2108. 34537440 10.1016/j.jtho.2021.07.035

[55] Soria JC, Tan DSW, Chiari R, et al. First-line ceritinib versus platinum-based chemotherapy in advanced ALK-rearranged non-small-cell lung cancer (ASCEND-4): a randomised, open-label, phase 3 study. Lancet. 2017;389(10072):917–929. 28126333 10.1016/S0140-6736(17)30123-X

[56] Solomon BJ, Besse B, Bauer TM, et al. Lorlatinib in patients with ALK-positive non-small-cell lung cancer: results from a global phase 2 study. Lancet Oncol. 2018;19(12):1654–1667. 30413378 10.1016/S1470-2045(18)30649-1

[57] Bauer TM, Shaw AT, Johnson ML, et al. Brain penetration of lorlatinib: cumulative incidences of CNS and Non-CNS progression with lorlatinib in patients with previously treated ALK-positive non-small-cell lung cancer. Target Oncol. 2020;15(1):55–65. 32060867 10.1007/s11523-020-00702-4 PMC7028836

[58] Shaw AT, Bauer TM, de Marinis F, et al. First-Line lorlatinib or crizotinib in advanced ALK-positive lung cancer. N Engl J Med. 2020;383(21):2018–2029. 33207094 10.1056/NEJMoa2027187

[59] Horn L, Wang Z, Wu G, et al. Ensartinib vs crizotinib for patients with anaplastic lymphoma kinase-positive non-small cell lung cancer: a randomized clinical trial. JAMA Oncol. 2021;7(11):1617–1625. 34473194 10.1001/jamaoncol.2021.3523 PMC8414368

